# High throughput RNAi assay optimization using adherent cell cytometry

**DOI:** 10.1186/1479-5876-9-48

**Published:** 2011-04-25

**Authors:** Christoph S Nabzdyk, Maggie Chun, Leena Pradhan, Frank W LoGerfo

**Affiliations:** 1 Division of Vascular and Endovascular Surgery, Beth Israel Deaconess Medical Center, Harvard Medical School, Boston, MA, USA

**Keywords:** RNAi, vascular, adherent cell cytometry, in vitro assay, high throughput

## Abstract

**Background:**

siRNA technology is a promising tool for gene therapy of vascular disease. Due to the multitude of reagents and cell types, RNAi experiment optimization can be time-consuming. In this study adherent cell cytometry was used to rapidly optimize siRNA transfection in human aortic vascular smooth muscle cells (AoSMC).

**Methods:**

AoSMC were seeded at a density of 3000-8000 cells/well of a 96well plate. 24 hours later AoSMC were transfected with either non-targeting unlabeled siRNA (50 nM), or non-targeting labeled siRNA, siGLO Red (5 or 50 nM) using no transfection reagent, HiPerfect or Lipofectamine RNAiMax. For counting cells, Hoechst nuclei stain or Cell Tracker green were used. For data analysis an adherent cell cytometer, Celigo^® ^was used. Data was normalized to the transfection reagent alone group and expressed as red pixel count/cell.

**Results:**

After 24 hours, none of the transfection conditions led to cell loss. Red fluorescence counts were normalized to the AoSMC count. RNAiMax was more potent compared to HiPerfect or no transfection reagent at 5 nM siGLO Red (4.12 +/-1.04 vs. 0.70 +/-0.26 vs. 0.15 +/-0.13 red pixel/cell) and 50 nM siGLO Red (6.49 +/-1.81 vs. 2.52 +/-0.67 vs. 0.34 +/-0.19). Fluorescence expression results supported gene knockdown achieved by using MARCKS targeting siRNA in AoSMCs.

**Conclusion:**

This study underscores that RNAi delivery depends heavily on the choice of delivery method. Adherent cell cytometry can be used as a high throughput-screening tool for the optimization of RNAi assays. This technology can accelerate *in vitro *cell assays and thus save costs.

## Background

RNAi technology is emerging as a promising tool for the treatment of cardiovascular disease. Various *in vitro *and *in vivo *studies have used siRNA to address the sequelae of vascular injury [1-3]. In order to provide a successful siRNA delivery, factors such as the choice of transfection reagent as well as the intrinsic susceptibility of the target cell type have to be evaluated prior to treatment. Additionally, endothelial and smooth muscle cells from the various segments of the circulation are known to have inherently different biological properties [4,5]. Recent work by Andersen et al. suggests that endothelial cells from human coronary artery display a higher susceptibility towards siRNA transfection than smooth muscle cells [6].

Under identical transfection conditions (siRNA concentration and sequence, transfection reagent, and mode of transfection) we found significant differences in gene knockdown achieved in primary human vascular smooth muscle cells from coronary artery compared to the aorta (data not shown). This might be in part due to the differential susceptibility of cells towards transfection reagents. Also, commercially available transfection reagents are numerous and their efficacy varies significantly between the individual cell types. Considering the multitude of variables, optimization of siRNA transfection, especially in less susceptible cells such as primary human aortic smooth muscle cells, can become time consuming and costly. Fluorescently labeled transfection indicators such as siGLO Red (Dharmacon Inc., Lafayette, CO) are helpful tools as they indicate the presence of siRNA within the target cell. The fluorescence signal emitted from the transfected cells serves as an indirect parameter for successful transfection. However, objective quantification of fluorescence signal intensity derived from transfected cells can be laborious when a large number of samples is analyzed using either flow cytometry or manual analysis of fluorescent pixel counts. In addition, flow cytometry analysis of vascular smooth muscle cells or other stromal cells is more difficult to perform compared to e.g., lymphocytes, due to the heterogeneous shape of stromal cells and the tendency to aggregate and clump.

Therefore it is desirable to have a system that provides a rapid high-through-put analysis of fluorescence expression in transfected cells to guide the transfection strategy. In the present study, the adherent cell cytometry system Celigo^® ^(Cyntellect Inc., San Diego, CA) was used; a bench top in situ system that rapidly generated whole well images and instant fluorescence based analysis. Ideally, this system would allow multiple cross comparisons between the different treatment conditions. Further, the availability of such a system might reduce the dependence on Q-RT-PCR and flow cytometry for the optimization of siRNA assays and thereby help reduce time and costs.

## Methods

For a detailed list of reagents and equipment used see Additional File 1.

### Cell Culture

Human Aortic Smooth Muscle Cells (Lonza, Walkersville, MD) were cultured in basal medium (LifeLine, Walkersville, MD) enriched with the supplied SMC growth additives. The media was maintained in a humidified incubator at 37°C with 5% CO2. Cells from passages 6 - 9 were used in the experiments.

### siRNA/siGLO Red transfection

**Figure 1:**
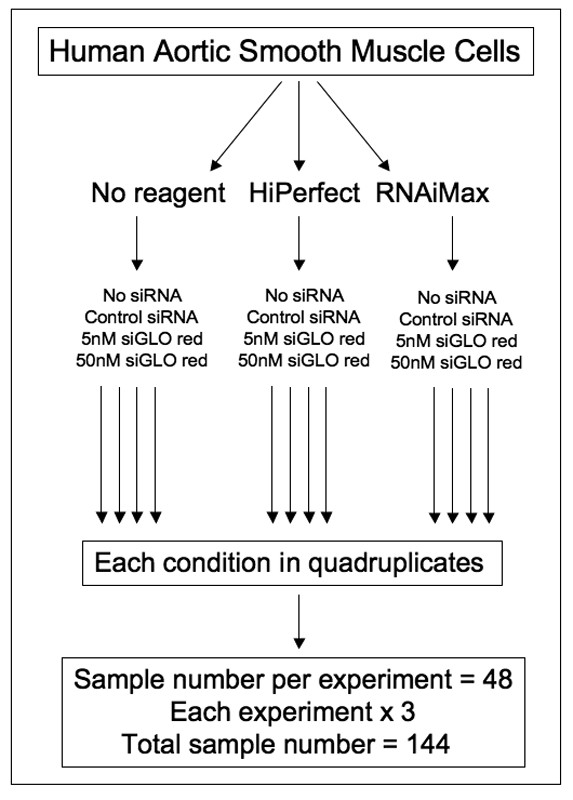
**Experimental conditions and total number of samples**. Human aortic smooth muscle cells were transfected in the presence or absence of a transfection reagent (HiPerfect™ or RNAiMax™; 0.375 μl/100 μl each). Each group was further divided into four conditions: no siRNA, 50 nM unlabeled control siRNA, and 5 nM and 50 nM of siGLO Red transfection indicator. Each treatment was carried out in quadruplicates and each experiment was repeated three times for a total sample size of 144.

Confluent AoSMCs were seeded at a density of 3000-8000 cells/well in a BD Falcon 96-well black-bottom plate (Fisher, Pittsburg, PA). 24 hours later, cells were transfected with either non-targeting unlabeled siRNA (50 nM) (CAT#ID D-001206-13-20, Dharmacon, Lafayette, CO), siRNA targeting human MARCKS (CAT#ID D-004772-04, Dharmacon, Lafayette, CO) or siGLO Red (5 or 50 nM) (Dharmacon, Lafayette, CO) using no transfection reagent, HiPerfect (Qiagen, Valencia, CA), or Lipofectamine RNAi Max (Invitrogen, Carlsbad, CA) as recommended by the manufacturer. A master mix was created for each individual condition in order to eliminate pipetting errors and to increase consistency between each well. The experimental set-up of the twelve conditions for each cell type is outlined in Figure 1.

### Plate analysis with the adherent cell cytometry system Celigo^®^

The system used in this study allows quantification of cellular responses in formats ranging from 1536- to 6-well plates and T-25 and T-75 flasks, both at full resolution of 1 mm/pixel as well as half-resolution (2 × 2 binning). The working aperture of the scan lens is 9 mm, and the system enables very rapid imaging by using fast galvanometer mirrors to scan and stitch multiple fields-of-view into a full resolution image, requiring far fewer mechanical stage movements and focus operations as compared with conventional microscopes. This design enables imaging of the entire well, including every cell in every well in the analysis. Image segmentation capabilities allow for customized analysis and a real-time gating interface to enable subpopulations of cells to be defined/quantified based on simple or complex phenotypes. Multi-parameter analysis can be based on morphology features (e.g. cell area, shape, etc.) and/or functional readouts (e.g. fluorescence expression markers, reporters).

Prior to plating, cells were stained with Cell Tracker Green (Invitrogen) and with Hoechst nuclei stain (2.6 μg/mL, Invitrogen). Plates were read using the adherent cell cytometer equipped with a brightfield and three fluorescent channels: a blue filter for the Hoechst nuclei stains, red filter for the siGLO Red, and green for the Cell Tracker Green cytoplasmic dye.

Gating parameters were adjusted for each fluorescence channel to exclude background and other non-specific signals. The Celigo^® ^system provided a gross quantitative analysis for each fluorescence channel, including total counts of gated events.

### RNA extraction and Q-RT-PCR

RNA was extracted and cDNA was generated using the Cells-to-CT™ Kit (Ambion, Foster City, CA). Q-RT-PCR was performed using Power Sybr Green Mastermix (Ambion, Foster City, CA) and a Stratagene™Mx3000p Q-RT-PCR system (Stratagene, La Jolla, CA) (PCR primers in Table 1). Gene knockdown was calculated using the ΔΔ-Ct method.

**Table 1:** List of Q-RT-PCR primers

Primer ID	Sequence 5'-3'
MARCKS forward	CGGCAGAGTAAAAGAGCAAGC

MARCKS reverse	GGTTGTAGACAAGTTCTCCAAAAC

B2M forward	CTCCACAGGTAGCTCTAGGAG

B2M reverse	TCTGACCAAGATGTTGATGTTGG

### Statistical Analysis

At least three independent experiments were performed and results were analyzed using Graph Pad Prism Version 5.0 software (Graph Pad Software Inc, La Jolla, CA). For expression analysis, two-way ANOVA with Bonferroni post-hoc analysis was used to assess statistical significance. For Q-RT-PCR analysis, one-way ANOVA with Bonferroni post-hoc analysis was performed. A 'p' value of less than 0.05 was considered as statistically significant.

## Results

### Cell count and cytotoxicity of transfection reagents

**Figure 2:**
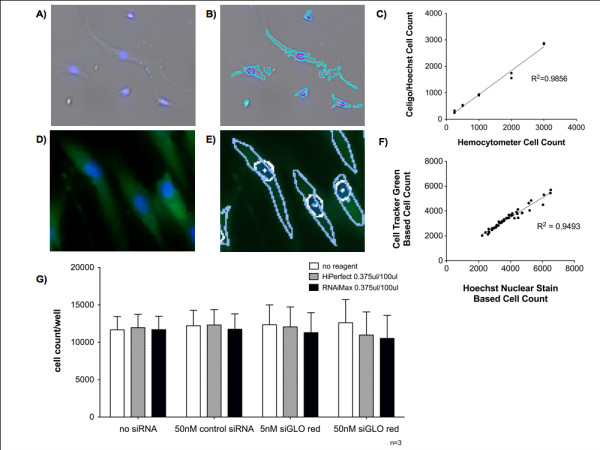
**Cell counts of live adherent human AoSMCs in cell culture using Celigo^® ^cytometer**. **(A) **Overlay of AoSMC brightfield and DAPI filter images (Hoechst nuclei stain, blue). **(B) **Segmentation of AoSMCs seen in **(A) **and their nuclei. For the detection specific gating parameters were adjusted for the Celigo^® ^cytometer. Although suboptimal brightfield segmentation (light blue borders) was observed when gating the individual AoSMCs, the Celigo^® ^system segmented the nuclei (purple borders) accurately due to their homogenous shape and strong fluorescence signal and thus provided a precise total cell count. **(C) **Correlation between cell counts obtained by a hemocytometer and the Celigo^® ^cell count based on Hoechst nuclei stain. **(D) **AoSMCs dual labeled with Hoechst nuclei stain and cytoplasmic dye Cell Tracker Green. **(E) **Overlay of fluorescence channels with gated nuclei (white line) and cytoplasm border (light blue line) overlapping. **(F) **Individual stain counts (blue, DAPI filter and green, FITC filter) compared in dual labeled cells with Hoechst nuclei stain (blue) and the cytoplasmic dye Cell Tracker Green (green). **(G) **AoSMC counts were not significantly different between the individual treatment conditions.

Fluorescence expression analysis and cell counts were obtained. The Celigo^® ^system allows basic assessment of size and morphology of live adherent cells based on brightfield contrast differences between the cell membrane, cytosol, and extracellular space. However, at higher cell densities and due to the complex geometry of AoSMCs, it was difficult to obtain exact cell counts using the Celigo^®^'s brightfield cell segmentation function (Figure 2A & 2B). In order to address this issue, AoSMCs were trypsinized and manually counted using a hemocytometer prior to plating. AoSMCs were then seeded at known densities into 96-well plates, allowed to attach, and then counted by automated fluorescence expression analysis using Hoechst nuclear stain and the system's gating function. A strong cell count correlation was observed between the two methods with r^2 ^= 0.9856 (Figure 2C).

Subsequently, cell counts based on Hoechst nuclear stain were compared side by side with counts based on a cytoplasmic stain (Cell Tracker Green, Molecular Probes) (Figure 2D-E). Both approaches allowed for reliable and comparable cell counts with a strong correlation between the two methods with r^2 ^= 0.9493 (Figure 2F).

In order to quantify cytotoxicity of our treatments, cell counts post-siRNA/siGLO Red transfections were assessed. No significant cell loss was observed 24 h after transfection in any of the treatment groups irrespective of the choice of transfection reagent or siRNA or siGLO Red concentration (p > 0.05) (Figure 2G).

### Transfection differences between transfection reagents in human primary aortic smooth muscle cells

**Figure 3:**
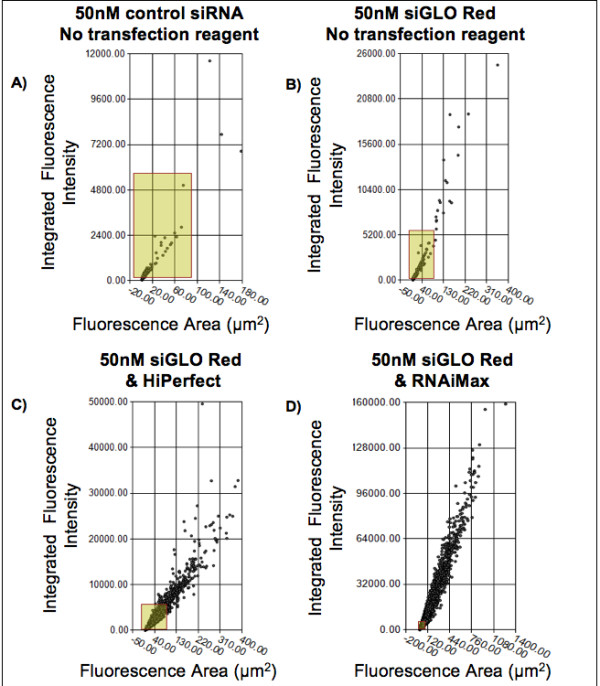
**Scatter plots of control siRNA or siGLO Red transfected AoSMCs using different transfection reagents**. Composition of scatter plot diagrams generated with Celigo^® ^system software. Parameters depicted are fluorescence area (x-axis) and integrated fluorescence intensity (y-axis). **(A) **50 nM unlabeled control siRNA, no transfection reagent group. The area boxed in yellow represents the majority of red signal fluorescence (background fluorescence) in this group. **(B) **50 nM siGLO Red, no transfection reagent group. The yellow highlighted area from (A) serves as a comparison. **(C)**50 nM siGLO Red with HiPerfect group. Boxed area of (A) compared to the gated events recorded in (C). **(D) **50 nM siGLO Red with RNAiMax group. Area of (A) compared to gated events recorded in AoSMCs transfected with 50 nM siGLO Red complexed with RNAiMax transfection reagent.

Celigo^® ^software was used to generate representative scatter plots that depict red fluorescence area (in μm^2^) and fluorescence integrated intensity (sum of the pixel intensities within a gated event) of the gated events in the individual treatment groups within a single well of the 96-well plate (Figure 3).

Minimal background fluorescence was detected in AoSMCs treated with 50 nM unlabeled control siRNA (no transfection reagent) as depicted in Figure 3A. Strong differences were seen in total count, fluorescence area size, and integrated intensity of red fluorescence signals in AoSMCs treated with 5 nM or 50 nM siGLO Red depending on the transfection reagent (5 nM data not shown, Figure 3B-D). The presence of either HiPerfect or RNAiMax drastically increased the transfection success compared to 'no transfection reagent'. Further, 50 nM siGLO Red combined with RNAiMax showed higher transfection rates compared to HiPerfect.

**Figure 4:**
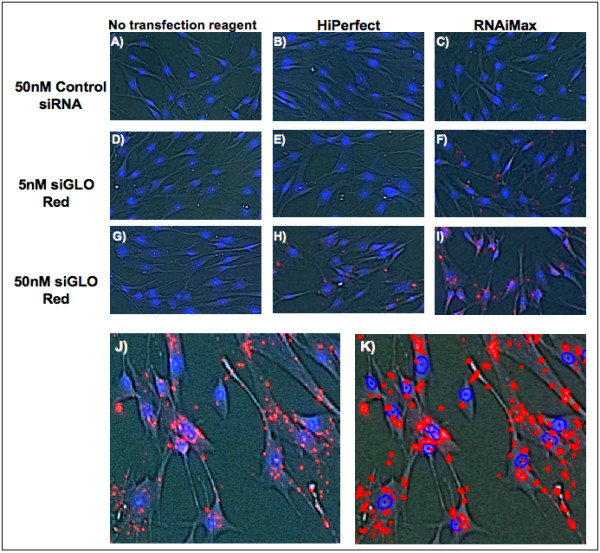
**Comparison of transfection reagents in siGLO Red transfected AoSMCs**. **(A, D, and G) **No transfection reagent with 50 nM control siRNA, 5 nM siGLO Red and 50 nM siGLO Red respectively. **(B, E, and H) **HiPerfect transfection reagent (0.375 μl/100 μl) with 50 nM control siRNA, 5 nM siGLO Red and 50 nM siGLO Red respectively. **(C, F, and I) **RNAiMax transfection reagent (0.375 μl/100 μl) with 50 nM control siRNA, 5 nM siGLO Red and 50 nM siGLO Red respectively. **(J) **Representativepicture of 50 nM siGLO Red + RNAiMax transfected AoSMCs used for quantification. AoSMCs were also stained with Hoechst nuclei stain. Both fluorescent signals were confined within the cells. **(K) **Represents Figure 4J with the gated fluorescent events (red dots/circles = siGLO Red; blue circles = Hoechst stained nuclei). Note the exact overlay of actual and detected fluorescent events. The vast majority of gated signals originated from the AoSMCs.

Visualization of the transfected cells confirmed that the vast majority of red fluorescence originated from siGLO Red transfected AoSMCs with only minimal extracellular signals (Figure 4A-I). In addition, the adherent cell cytometer precisely detected the nuclei and fluorescently labeled siGLO Red siRNA within the AoSMCs (Figure 4J and 4K).

Next we normalized the total red fluorescence pixel count (not accounting for fluorescence intensity or area) per well to the cell count in the individual well in order to control for variations in cell seeding densities. Subsequently, the fraction of recorded red fluorescence pixel divided by the nuclei number was termed 'red pixel per cell' or 'red pixel/nucleus.'

**Figure 5:**
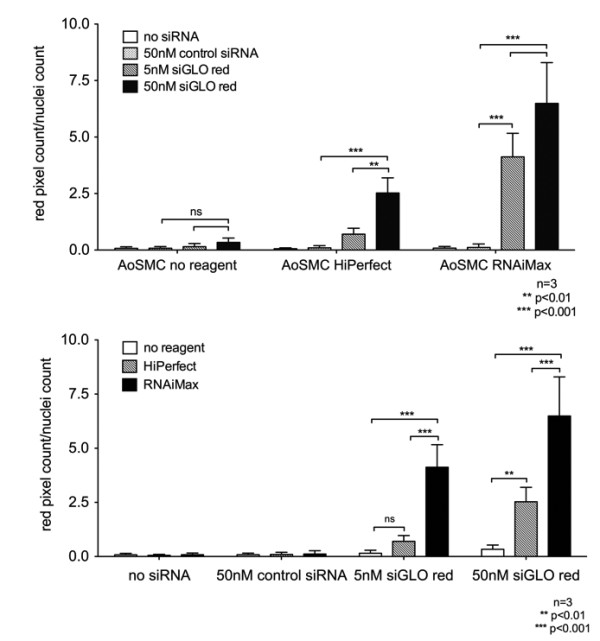
**Comparison of siGLO Red counts per cell in different treatment conditions**. **(A) **Comparison of siGLO Red pixel counts/cell among different siRNA treatment conditions in AoSMCs that have been transfected without transfection reagent, or with HiPerfect, or RNAiMax. **(B) **Transposition of (A). Comparison of siGLO Red pixel count/cell among different transfection reagents in AoSMCs that have either not been transfected or were transfected with 50 nM control siRNA, 5 nM siGLO Red or 50 nM siGLO Red.

Background fluorescence was negligible in all unlabeled control groups (no siRNA and 50 nM unlabeled control siRNA) and no significant differences were detected between the individual groups irrespective of the transfection reagent (Figure 5).

In the fluorescent labeled siGLO Red treated cells, all transfection reagent groups showed a dose dependent increase (5 nM and 50 nM siGLO Red) in fluorescence signal count per cell compared to unlabeled control groups (Figure 5).

Interestingly, there was no difference in fluorescence pixel count/cell when AoSMCs were treated with 5 nM siGLO Red and HiPerfect compared to AoSMCs transfected with 5 nM siGLO Red and no transfection reagent. Significant differences were observed only when 50 nM siGLO Red and HiPerfect were used when compared to no transfection reagent.

However, when AoSMCs were treated with RNAiMAX and siGLO Red, fluorescence pixel counts/cell were significantly higher compared to AoSMCs transfected with siGLO Red using either HiPerfect or no transfection reagent. This was observed in AoSMCs transfected with 5 nM siGLO Red (RNAiMax vs. HiPerfect vs. no transfection reagent; 4.12 +/-1.04 vs. 0.70 +/-0.26 vs. 0.15 +/-0.13 red pixel/cell, respectively) as well as in the 50 nM siGLO Red groups (6.49 +/-1.81 vs. 2.52 +/-0.67 vs. 0.34 +/-0.19 red pixel/cell, respectively).

**Figure 6:**
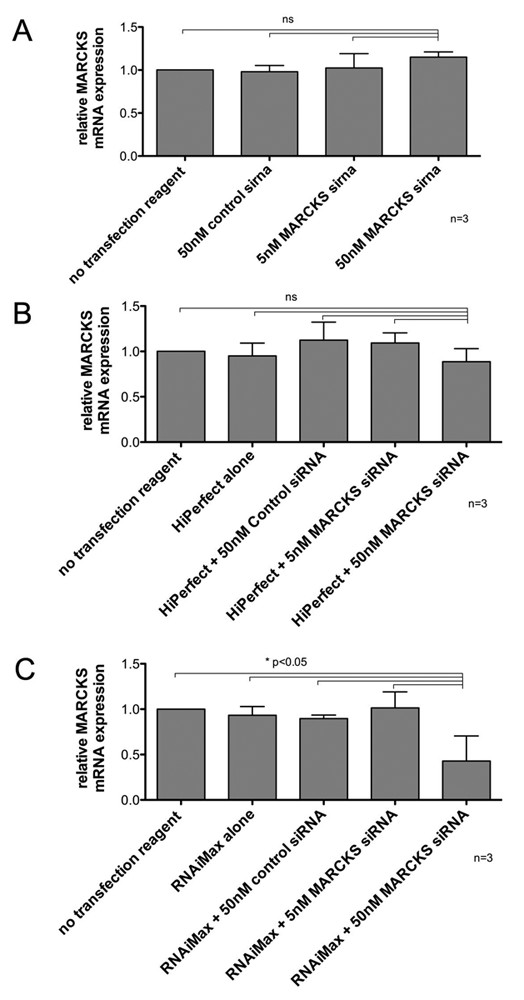
**MARCKS silencing in AoSMCs**. (A) No transfection reagent was used. No significant knockdown was achieved using 5 nM or 50 nM MARCKS compared to no reagent control. (B) Transfection reagent HiPerfect combined with 5 nM or 50 nM MARCKS siRNA did not result in significant MARCKS gene knockdown compared to control groups. (C) RNAiMax combined with 50 nM MARCKS siRNA resulted in a significant reduction of MARCKS mRNA levels compared to control groups.

In order to correlate fluorescence expression findings with actual gene silencing, AoSMCs were transfected with siRNA targeting the gene MARCKS. The MARCKS siRNA protocol had been established prior to this study in our lab [6]. Levels of gene knockdown were analyzed using Q-RT-PCR. With the exception of exchanging siGLO Red with MARCKS siRNA, identical transfection conditions were used as in the adherent cytometer experiments. Q-RT-PCR data supported siGLO Red transfection experiments. MARCKS siRNA in the absence of a transfection reagent or complexed with HiPerfect did not lead to a significant MARCKS gene knockdown compared to control (Figure 6A and 6B). Only 50 nM MARCKS siRNA complexed with RNAiMax led to a significant reduction of MARCKS mRNA levels by 60% compared to controls (Figure 6C). In accordance with the Q-RT-PCR results, 50 nM siGLO Red complexed with RNAiMax provided significantly higher transfection rates (red fluorescence pixel/per cell) than any other transfection condition.

## Discussion

RNAi technology is a powerful and promising new tool in the field of gene therapy.

However, in order to achieve the highest possible biological effect, several aspects of siRNA delivery must be addressed and adapted to the individual experimental model.

Given the vast amount of cell lines and primary cells available, combined with the long list of transfection reagents, it can be time consuming and expensive to identify the optimal transfection conditions for the experiment. Fluorescence-based transfection indicators such as siGLO Red are helpful guides. However, quantification of fluorescence intensity is labor intensive if performed manually using imaging software. Alternatively, flow cytometry systems require large cell numbers and hence increase experimental costs. Additionally, primary stromal cells such as AoSMCs are difficult to transfect and analyze using flow cytometry. Therefore it is desirable to establish an assay that allows for a high throughput analysis of adherent cells based on fluorescence expression. Our data demonstrate that adherent cell cytometry provides ample opportunities to customize cellular assays in vitro and can help accelerate experimental optimization in a high throughput fashion e.g. in the setting of siRNA transfection. Due to the design of the adherent cytometry systems, cell numbers and reagent volumes can be minimized compared to flow cytometry, reducing experimental costs and allowing for multiple replicates and various comparisons on an individual plate.

In this exemplary study, the efficacy of two commercially available transfection reagents (HiPerfect, RNAiMax) in combination with two different concentrations of siGLO Red transfection indicator (5 and 50 nM) or 50 nM unlabeled control siRNA was examined.

Transfection success was measured by the number of red fluorescent signals representing the transfection indicator siGLO Red within the AoSMCs. This count was then normalized to the cell count in the analyzed well. We analyzed AoSMCs after transfection with either 'no siRNA,' '50 nM unlabeled control siRNA,' '5 nM siGLO red,' or '50 nM siGLO red' using either 'no transfection reagent,' 'HiPerfect,' or 'RNAiMax.'

Results showed that the Celigo^® ^system can be conveniently and accurately used to count live cells based on the two live cell dyes, Hoechst or Cell tracker green, with a strong correlation between the two methods.

Neither siRNA nor siGLO Red treatment led to cell loss 24 h post-transfection. Only minimal fluorescence signals were observed in the 'no transfection reagent' group even when using a high siGLO Red concentration. Presence of HiPerfect and RNAiMax increased rate of transfection compared to no transfection reagent when combined with 5 and 50 nM siGLO Red. RNAiMax provided a higher rate of transfection compared to HiPerfect both at 5 nM (0.70 versus 4.12 red pixels/cell, respectively) and 50 nM siGLO Red (2.52 versus 6.49 red pixels/cell, respectively). These findings helped our research as we identified RNAiMax as the more potent transfection reagent of the two, a relevant finding for our subsequent experiments. Importantly, fluorescence expression results were supported by the matching Q-RT-PCR data from experiments in which the gene MARCKS was silenced. Q-RT-PCR data revealed that only 50 nM MARCKS siRNA (in place of siGLO Red) complexed with RNAiMax led to a significant reduction of MARCKS mRNA levels of 60% in AoSMCs while all other conditions did not. According to the adherent cytometer data, successful siGLO Red transfections were also achieved when transfecting AoSMCs with siGLO Red (5 and 50 nM) complexed with HiPerfect and 5 nM siGLO Red with RNAiMax. However, these findings were not exactly mirrored in the corresponding Q-RT-PCR experiments.

This could be due to the necessity of a critical level of intracellular siRNA in order to facilitate significant gene silencing. Other explanations could be that some of the detected siGLO Red was attached to the outside of the cell or trapped within the cell membrane and thus did not enter the RISC complex. Further, once siRNA enters the cell it has to escape the endosome ('endo-lysosomal escape') in order to hybridize with the target mRNA. It is possible that some of the detected intracellular siGLO Red was trapped within an endo-lysosome and therefore also prevented it from entering the RISC complex. Given the discussed biological barriers that the siRNAs need to overcome, it is not surprising that a significant gene knockdown only occurred at higher transfection rates (red pixel/cell). In our experiments this threshold was surpassed only in the 50 nM MARCKS siRNA and RNAiMax treatment group. Taken together our data suggest that adherent cell cytometry is a very sensitive method that can detect spurious amounts of fluorescently labeled siRNA localized on the outside or within the target cells.

More importantly, the analysis of an individual experiment with 48 samples in a 96-well plate using the Celigo^® ^system only took about 30 minutes. This analysis consisted of cell count and fluorescence expression analysis (total counts, area, mean, and integrated fluorescence intensity). It is safe to assume that it would have taken a much longer time to obtain the same cell count using either a hemocytometer or an automated cell counter. Colorimetric assay such as the Alamar Blue assay, although commonly used to assess proliferation and cytotoxicity, is not very sensitive and cell counts obtained from this assay are affected by the metabolic state of the cells tested.

Our data show that adherent cell cytometry is a versatile technology that can be used to analyze customized assays such as fluorescence-based cell function assays, either at one specific time point or over repeated time points. In fact, results could also be obtained from fixed and immunofluorescence labeled cells. With the availability of three fluorescence channels in the adherent cell cytometer, one channel can be used to detect the fluorescently labeled siRNA and the other two can be used to identify the effects of intracellular siRNA on cellular functions using live cell fluorescence reporter assays. For example, this set-up could be used to screen siRNA libraries to identify the siRNA sequence that exerts the strongest effects on cell proliferation or apoptosis. Another benefit of the system is the simultaneous assessment of various cell functions within live adherent cells without interrupting cell-cell contacts. The bench-top format of this technology may significantly accelerate *in vitro *experiments involving adherent cells for non-industry related laboratories.

## Conclusion

Adherent cytometry is a versatile technology that can be used to monitor proliferation, cell death and other cell functions in live cells. In this study we demonstrated that adherent cell cytometry is a simple method to detect fluorescently labeled siRNA in target cells. Based on fluorescence indicator expression analysis our results suggest that there are significant differences in the efficacy of the transfection reagents HiPerfect and RNAiMax, with RNAiMax showing higher transfection rates in human AoSMCs. Matching Q-RT-PCR data support these findings. Taken together, our data suggest that adherent cell cytometry can be used as a high throughput-screening tool for the optimization of RNAi assays. This technology can accelerate various *in vitro *cell assays and thus save time and costs.

## Competing interests

The authors declare that they have no competing interests.

## Authors' contributions

CSN - Experimental design, data acquisition and analysis, editing of the manuscript. MC - Data acquisition, editing of the manuscript. LP - Experimental design, manuscript review. FWL - Experimental design, manuscript review. All authors read and approved the final manuscript.

## Supplementary Material

Additional file 1 **List of reagents and equipment**. Comprehensive list of reagents and equipment used for the described experiments including catalogue numbers. Click here for file
