# Supplementary material for: High throughput RNAi assay optimization using adherent cell cytometry
Source: J Transl Med. 2011 Apr 25;9:48. doi: 10.1186/1479-5876-9-48 (PMC3111359; doi:10.1186/1479-5876-9-48)
Supplement: Additional file 1 — List of reagents and equipment. Comprehensive list of reagents and equipment used for the described experiments including catalogue numbers. [file 1479-5876-9-48-S1.DOC]

**SUPPLEMENTAL INFORMATION**

**List of reagents and equipment**

| **Materials** |  |  |  |
| --- | --- | --- | --- |
|  |  |  |  |
| **Cells** | **Vendor** | **City, State** | **Catalogue #** |
| Human Aortic Smooth Muscle Cells | Lonza | Walkersville, MD | CC-2571 |
| Smooth muscle media complete kit | Life Line | Walkersville, MD | LL-0014 |
|  |  |  |  |
| **Fluorescent Stains/siRNA** |  |  |  |
| Hoechst Nuclei Stain | Invitrogen | Carlsbad, CA | H3570 |
| siGLO Red Transfection Indicator (5nmol) | Dharmacon | Lafayette, CO | D-001630-02-05 |
| siGenome Control Pool (50uM) | Thermo Scientific | Lafayette, CO | D-001206-13-20 |
| Invitrogen Cell Tracker Green (50ug) | Invitrogen | Carlsbad, CA | C7025 |
|  |  |  |  |
| **Transfection Reagents** |  |  |  |
| HiPerfect | Qiagen | Valencia, CA | 301702 |
| Lipofectamine RNAiMax | Invitrogen | Carlsbad, CA | 13778075 |
|  |  |  |  |
| **Cell Culture Plate** |  |  |  |
| BD Falcon 96-well Black-Bottom Plates | Fisher | Pittsburg, PA | 353948 |
|  |  |  |  |
| **Plate reading Instrument** |  |  |  |
| Celigo adherent cell cytometer | Cyntellect | San Diego, CA |  |
